# Acquisition of the *lac* operon by *Salmonella enterica*

**DOI:** 10.1186/s12866-015-0511-8

**Published:** 2015-08-25

**Authors:** Susan R. Leonard, David W. Lacher, Keith A. Lampel

**Affiliations:** Division of Molecular Biology, Center for Food Safety and Applied Nutrition, U.S. Food and Drug Administration, Laurel, MD USA

## Abstract

**Background:**

Classical bacteriological characteristics of *Salmonella enterica* indicate that the members of this species are unable to utilize lactose as a carbon source. However, lactose-fermenting (Lac+) strains of several *Salmonella* serovars have been isolated from different foodborne outbreaks as well as different geographical regions worldwide. In the present study, we sequenced the genomes of 13 Lac + *S. enterica* isolates and characterized the *lac* region, comparing it to the *lac* region in other enteric bacterial species.

**Results:**

Genetic analysis of the *lac* operons in the *S. enterica* genomes revealed that they all contain intact *lacI, lacZ,* and *lacY* genes. However, *lacA* was truncated in all of the *S. enterica* subsp. *enterica* isolates, encoding a 56 amino acid peptide rather than the full length 220 amino acid LacA protein. Molecular analyses of the 13 isolates revealed that the *lac* operon resided on a plasmid in some strains and in others was integrated into the bacterial chromosome. In most cases, an insertion sequence flanked at least one end of the operon. Interestingly, the *S. enterica* Montevideo and *S. enterica* Senftenberg isolates were found to harbor a plasmid with a high degree of sequence similarity to a plasmid from *Klebsiella pneumoniae* strain NK29 that also harbors the *lac* operon. In addition, two *S. enterica* Tennessee isolates carried two copies of the *lac* operon. Phylogenetic analysis based on *lacIZY* gene sequences determines distinct clusters, and reveals a greater correlation between *lacIZY* sequence and flanking organization than with either bacterial species or genomic location.

**Conclusions:**

Our results indicate that the *lac* region is highly mobile among *Enterobacteriaceae* and demonstrate that the Lac + *S. enterica* subsp. *enterica* serovars acquired the *lac* region through parallel events. The acquisition of the *lac* operon by several *S. enterica* serovars may be indicative of environmental adaptation by these bacteria.

**Electronic supplementary material:**

The online version of this article (doi:10.1186/s12866-015-0511-8) contains supplementary material, which is available to authorized users.

## Background

*Salmonella enterica* consists of over 2500 serovars, most of which are non-typhoidal and invade the host intestinal epithelium resulting in enterocolitis/diarrhea [1, 2]. Transmission of non-typhoidal *S. enterica* occurs predominately through contaminated food or water, but also spreads by person to person contact, or contact with infected animals [1, 2]. Lactose-fermenting ability is a biochemical test often used diagnostically to differentiate *S. enterica* from other *Enterobacteriaceae*, particularly *Escherichia coli*, as the genes responsible for conferring the lactose-fermenting phenotype are not harbored by most *S. enterica* strains [3, 4]. However, although infrequent, outbreaks caused by lactose-fermenting (Lac+) *S. enterica* have been reported [5–7], including a recent outbreak due to contamination of peanut butter with *S. enterica* Tennessee that sickened 715 individuals [8]. Occasional incidences of Lac + *S. enterica* have been reported for a variety of serovars [9–16].

The *lac* operon, comprised of the genes *lacZ, lacY,* and *lacA*, encodes the proteins responsible for lactose utilization. LacY, lactose permease, transports lactose into the cytoplasm where LacZ, β-galactosidase, cleaves lactose into glucose and galactose. The *lacA* gene encodes a transacetylase that is not an essential requirement for lactose catabolism. The *lac* region also includes the *lacI* gene, located just upstream of *lacZ*, that encodes a repressor that regulates transcription of the *lac* operon. While the *lac* region is located in the *E. coli* chromosome, it has been reported to be carried on a plasmid in several *Enterobacteriaceae* including two serovars of *S. enterica* [12]. Cornelis et al. determined that the *lacI*, *lacZ*, and *lacY* genes, along with flanking sequence, are found on a plasmid-encoded transposon, Tn951, in a Lac + strain of *Yersinia enterocolitica*. [17]. Isolation of a Lac + plasmid from *S. enterica* Typhi has been reported and an insertion sequence (IS) element, IS1, was revealed to be in the vicinity of the *lac* region on the plasmid [14]. However, in general, only phenotypes have been reported for Lac + *S. enterica* with no accompanying sequence or genetic analysis.

Horizontal transfer of transmissible elements has played a major role in the acquisition of new genetic information by bacterial species. The underlying physiological explanations for the acquisition or loss of genetic information, such as the *lac* operon, are not always apparent. Loss of genetic information may enhance virulence, and in other situations, would improve the bacterium’s ability to adapt to its environment (host). Characterization of the *lac* region could aid in understanding how the *lac* operon is transferred between enteric bacteria, including Lac + *S. enterica* isolates. In the current study, we compare the *lac* region and flanking sequence of the genome in 13 lactose-fermenting *S. enterica* isolates belonging to five different serovars along with other enteric bacterial species. Although a number of Lac + *Salmonella* strains have been identified since the turn of the 20^th^ century [11], a detailed comparison of this region in representatives in this genus has not been reported. Several of the isolates chosen in this study either originated in regions where Lac + *Salmonella* are more common (*S. enterica* Montevideo) or isolated from a food-related outbreak (*S. enterica* Tennessee).

To elucidate a potential transfer mechanism for the *lac* genes, the regions that surround the *lac* operon were sequenced to identify any genetic elements that may be involved in the mobilization of these genes. We demonstrated that with the exception of the *S. enterica* subsp. *diarizonae* isolates, the *lac* region is flanked by insertion sequences and that this entire genetic region is itself carried within a mobile genetic element. Whole genome sequence analyses in this study provide a putative mechanism as to the gain of the *lac* operon via transposable elements. Acquisition of these additional genes may provide the host bacterium, i.e., Lac + *Salmonella,* advantages to environmental adaptation.

## Results

### Molecular serotyping

The serotypes of the *S. enterica* isolates were confirmed by molecular means and are listed in Table 1. Two of the isolates were identified as *S. enterica* subsp. *diarizonae*. The lactose-fermenting ability exhibited by these isolates is not surprising since 85 % of *S. enterica* subsp. *diarizonae* are known to be Lac + [18], a much higher percentage than the 0.8 % reported for *S. enterica* subsp. *enterica* [4]. The remaining 11 isolates are serovars of *S. enterica* subsp. *enterica*, including five Senftenberg, three Tennessee, two Montevideo, and one Indiana. While a Lac + phenotype has been reported occasionally for a variety of *S. enterica* subsp. *enterica* serovars [5–9, 12–16], to our knowledge, lactose-fermenting Montevideo strains have not been reported previously.

**Table 1:** Genetic organization and genomic location of *lac* and flanking region for *Salmonella* isolates and other *Enterobacteriaceae*

Accession no.	Isolate	Location	5'	*algL* ^b^	*lacIZY*	*lacA* ^c^	3'
JZTM00000000	*S. enterica* Montevideo 50262	plasmid	IS903D	+	+	t	IS1
JZTT00000000	*S. enterica* Montevideo 50270	plasmid	IS903D	+	+	t	IS1
JZTN00000000	*S. enterica* Senftenberg 50263	plasmid	IS903D	+	+	t	IS1
JZTP00000000	*S. enterica* Senftenberg 50265	plasmid	IS903D	+	+	t	IS1
JZTU00000000	*S. enterica* Senftenberg 50271	plasmid	IS903D	+	+	t	IS1
JZTV00000000	*S. enterica* Senftenberg 50272	plasmid	IS903D	+	+	t	IS1
JZTO00000000	*S. enterica* Senftenberg 50264	plasmid	IS903D	+	+	t	IS1
EF382672	*K. pneumoniae* pK29	plasmid	IS903D	+	+	t	IS1
JZTS00000000	*S. enterica* Indiana 50269	chromosome	transposon	++	+	t	IS1
CP006731	*C. sakazakii* CMCC 45402	chromosome	IS5075	++	+	t	IS1
CP010377	*E. cloacae* 34983	chromosome	IS5075	++	+	t	IS1
CP004091	*C. sakazakii* SP291	chromosome	ISEhe3^a^	-	+	t	IS1^a^
CP001918	*E. cloacae* ATCC 13047	chromosome	ISEhe3^a^	-	+	t	IS1^a^
CP008842	*K. oxytoca* pKOXM1A	plasmid	IS26	+^a^	+	-	IS903D
CP006927	*K. pneumoniae* p30660_1	plasmid	IS5075	++	+	-	IS903D^a^
CP000648	*K. pneumoniae* pKPN3	plasmid	IS5075	++	+	-	IS903D
JX442974	*K. pneumoniae* pKN-LS6	plasmid	ISEc8^a^	++	+	-	IS903D
JN233704	*K. pneumoniae* pKPN-IT	plasmid	ISEc8^a^	++	+	-	IS903D
CP006657	*K. pneumoniae* p1	plasmid	ISEc8^a^	++	+	-	IS903D
FO834905	*K. pneumoniae* pKP52.145_II	plasmid	IS2	++	+	t	IS1^a^
JX424424	*K. pneumoniae* pKPN_CZ	plasmid	IS5075	++	+	t	IS1
KF719971	*K. pneumoniae* pKP007	plasmid	ISEc8^a^	++	+	t	IS1
CP008843	*K. oxytoca* pKOXM1B	plasmid	iso-IS1	-	+	-	IS5
HF571988	*Y. enterocolitica* YE53/03	chromosome	IS2	-	+	t	IS1
JZTK00000000	*S. enterica* Tennessee 50260 *lac*2	chromosome	ISEc8^a^	++	+	t	IS1
JZTL00000000	*S. enterica* Tennessee 50261 *lac*2	chromosome	ISEc8^a^	++	+	t	IS1
JZTJ00000000	*S. enterica* Tennessee 50259	chromosome	*umuD*	-	+	t	IS1
JZTK00000000	*S. enterica* Tennessee 50260 *lac*1	chromosome	*umuD*	-	+	t	IS1
JZTL00000000	*S. enterica* Tennessee 50261 *lac*1	chromosome	*umuD*	-	+	t	IS1
AHUY01000000	*S. enterica* Tennessee 4535	chromosome	*umuD*	-	+	t	IS1
CP009855	*E. cloacae* pENT-22e	plasmid	ISEc8^a^	++	+	t	IS1
CP009866	*Pantoea* sp. PSNIH2	chromosome	acetyltransferase	-	+	t	IS1
BA000007	*E. coli* Sakai	chromosome	*mhpR*	-	+	+	*cynX*
AE014075	*E. coli* CFT073	chromosome	*yaiL*	-	+	+	*codA*
FN554766	*E. coli* 042	chromosome	*mhpR*	-	+	+	*cynX*
CP003289	*E. coli* 2011C-3493	chromosome	*mhpR*	-	+	+	*cynX*
U00096	*E. coli* K-12 MG1655	chromosome	*mhpR*	-	+	+	*cynX*
CP000880	*S. enterica arizonae* RSK2980	chromosome	*acnB*	-	+	+	*speD*
JZTQ00000000	*S. enterica diarizonae* 50267	chromosome	*acnB*	-	+	+	*speD*
JZTR00000000	*S. enterica diarizonae* 50268	chromosome	*acnB*	-	+	+	*speD*

^a^remnant

^b^alginate lyase gene upstream of *lacI*: 897 bp, +; 936 bp, ++; negative, -

^c^truncated, t; negative, -; positive, +

### Lac phenotype

MacConkey agar plates were examined for red colonies indicating lactose fermentation. All of the *S. enterica* isolates included in this study displayed a Lac + phenotype, demonstrating that the isolates lacking *lacA* retain functional *lac* operons.

### Characterization of the *lac* operon and flanking regions

The *lac* operon regions were analyzed for the presence of the four *lac* genes as well as the genetic elements that flank the *lac* region. The results of this analysis are shown in Table 1. Interestingly, it was discovered that the genomes of two of the Tennessee isolates, 50260 and 50261, harbor two *lac* regions. To distinguish the two *lac* operons for these isolates, they will be referred to as *lac*1 and *lac*2. All 13 *S. enterica* genomes were found to contain intact *lacI, lacZ,* and *lacY* genes. The *S. enterica* subsp. *diarizonae lac* regions also contained intact copies of the *lacA* gene. However, *lacA* was truncated in all of the *S. enterica* subsp. *enterica* isolates, encoding a 56 amino acid product rather than the full length 220 amino acid LacA found in *E. coli* K-12 MG1655. With the exception of the *E. coli* isolates, this discovery held for several other enteric bacteria examined in this study where *lacA* was either truncated or completely missing.

Extending the genetic analysis of the *S. enterica* subsp. *enterica* isolates to regions adjacent to the *lac* region revealed the presence of IS elements both upstream of *lacI* and downstream of the *lacA* remnant (Table 1). Characterization of the *lac* operon and flanking regions of other enteric bacterial species revealed that although in some cases the regions include different IS elements, *lac* operon regions are in almost all cases flanked by IS elements. Exceptions were the *S. enterica* Tennessee isolates whose genomes do not appear to contain an IS element upstream of the *lac* region (*lac*1 region of isolates 50260 and 50261) and the *E. coli* isolates that do not harbor IS elements at either end of the *lac* region. All of the *S. enterica* subsp. *enterica* isolates included in this study possess an IS1 element downstream of the *lacA* remnant, however, different IS elements are found at the 3' junction of some of the other species included in this study. The type of IS element located at the 5' junction of the *lac* region is more variable. It appears that the *lac* region is carried on several different transposable elements among *Enterobacteriaceae*. It was determined that the *lac* region found in the genomes of the *S. enterica* subsp. *diarizonae* isolates is not flanked by IS elements on either side.

Interestingly, characterization of the genome upstream of the *lac* region revealed an additional gene transcribed in the opposite direction of the *lac* genes between the IS element and *lacI* for the *S. enterica* isolates included in this study except isolate 50259, the *lac*1 region in isolates 50260 and 50261, and the *S. enterica* subsp. *diarizonae* isolates. This gene was found in the same genetic location in some of the other enteric bacterial species included in this study. BLASTp analysis revealed that the protein encoded by this gene has 99 % sequence identity to alginate lyase [GenBank:WP_012477405] encoded in the *Klebsiella pneumoniae* genome. For three of the *S. enterica* isolates, 50260, 50261, and 50269, this gene is 39 bp longer and the protein includes an additional oxidoreductase region, resulting in the highest protein sequence identity to a bifunctional oxidoreductase/alginate lyase found in *Enterobacteriaceae* [GenBank:WP_032414406]. The presence or absence of this additional gene along with the gene length if present is noted in Table 1.

### Genomic location of *lac* operon

We sought to determine where the mobile elements carrying the *lac* region were inserted in the *S. enterica* genomes. The *lac* regions of several of the *S. enterica* isolates in this study displayed a high sequence similarity and flanking organization with that of the *lac* region on plasmid pK29 harbored in *K. pneumoniae* strain NK29 [GenBank:EF382672]. Plasmid pK29 is 269,674 bp in size and the flanking organization is included in Table 1. The sequencing reads from the 11 *S. enterica* subsp. *enterica* isolates in this study were mapped to the plasmid pK29 reference sequence. The mappings demonstrated that seven of the isolates, namely Montevideo isolates 50262 and 50270 and Senftenberg isolates 50263, 50264, 50265, 50271 and 50272, harbor a plasmid with similarity to pK29. The consensus sequences resulting from the mappings revealed 99 % identity to pK29. Furthermore, BLAST comparisons revealed that these seven strains contain the *repHI2* and *repA* genes that encode replication proteins found in the pK29 plasmid sequence. Gaps in the *S. enterica* plasmid sequences were primarily due to the presence of regions containing a total of six antibiotic resistance genes in pK29. However, isolate 50271 was missing only three of the resistance genes. Although it was determined that the consensus from the mapping was ~20.8 kb shorter than pK29, it is not known whether there are additional insertions in the plasmids harbored by these seven *S. enterica* isolates.

The *lac* region of Tennessee isolate 50259 and the *lac*1 regions of Tennessee isolates 50260 and 50261 exhibited identical flanking organization and sequence with that of the *lac* region harbored on the chromosome of the *S. enterica* Tennessee isolate responsible for an outbreak associated with contaminated peanut butter [GenBank:AHUY01000000]. Genome comparisons confirmed that the *lac* regions harbored in the Tennessee isolates included in this study (*lac*1 regions for 50260 and 50261) are carried in the chromosome at the same location, within a 72 kb island inserted at *pheV*, as the Tennessee peanut butter outbreak isolate. Despite the similarity in flanking organization between the *lac*2 regions harbored in the genomes of isolates 50260 and 50261 with those found on two different plasmids, pKP007 and pENT-22e, carried in other enteric species (Table 1), the *lacIZY* sequences are not similar to those on the plasmids and sequencing read mappings determined that both isolates lack these plasmids. The *lac*2 regions appear to be located on the chromosome in an unknown location as both plasmid extractions and BLAST searches of the genome sequences for an origin of replication failed to reveal the presence of a plasmid in these isolates. The *lac* region was determined to be chromosomal for the Indiana isolate 50269. It is located within a 95 kb island inserted in the *ygiR* gene. Also chromosomally located, the *lac* region found in the *S. enterica* subsp. *diarizonae* isolates is inserted between the *acnB* and *speD* genes.

### Phylogenetic analysis of the *lacIZY* region

**Fig. 1:**
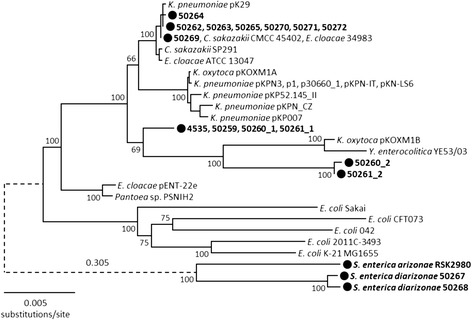
Phylogenetic relationships of *lacIZY* for *Salmonella* isolates and other *Enterobacteriaceae*. This neighbor-joining tree was constructed using the Kimura two-parameter model of nucleotide substitution. Bootstrap values based on 500 replications are given at the internal nodes. *Salmonella* isolates are indicated by the black circles and bold text. The branch connecting the *S. enterica* subsp. *arizonae/diarizonae* cluster with the rest of the isolates was shortened to improve the clarity of the relationships and its actual length is given above the dashed line

Along with characterizing the *lac* regions and flanking sequence in the genomes, we determined the sequence similarity of the concatenated *lacI, lacZ,* and *lacY* genes of the *S. enterica* isolates and other enteric bacterial species (Fig. 1). As expected, the *lac* genes carried by the Senftenberg and Montevideo isolates (50262-50265, 50270-50272) displayed a high degree of similarity (99.98 %) with those found on the *K. pneumoniae* plasmid pK29 (Additional file 1 Figure S1). In general, similar *lac* region flanking organization corresponds to highest *lac* gene sequence identity. One notable exception is the *lac*2 region in the Tennessee isolates 50260 and 50261 where the flanking IS elements, as well as the lengths of the gene upstream of *lacI* and the *lacA* remnant, are similar to the *K. pneumoniae* plasmid pKP007, but the *lacIZY* gene sequences share only 98 % identity. The *S. enterica* subsp. *diarizonae lac* gene sequences share low (73 %) homology with those of the *S. enterica* subsp. *enterica* serovars and other *Enterobacteriaceae*. Clearly, the *lac* gene sequences do not cluster according to plasmid or chromosomal location (Table 1, Fig. 1).

### MLST analysis of *Salmonella* isolates

**Fig. 2:**
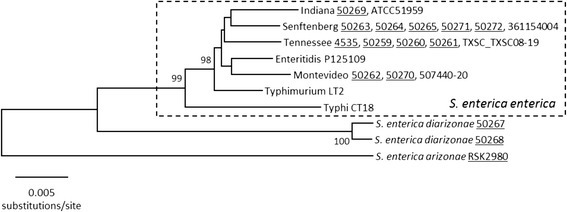
MLST analysis of *Salmonella* isolates. This neighbor-joining tree was constructed using the Kimura two-parameter model of nucleotide substitution. Bootstrap values >90 % based on 500 replications are given at the internal nodes. *S. enterica* subsp. *enterica* isolates are enclosed by the dashed box. Lac + isolates are underlined. The Lac- closed genome strains (Enteritidis P125109, Typhimurium LT2, and Typhi CT18) are included for reference

MLST analysis of the *Salmonella* isolates was performed using the *aroC*, *dnaN*, *hemD*, *hisD*, *purE*, *sucA*, and *thrA* loci (Fig. 2). Isolates belonging to the same serovar had identical sequence types (STs) regardless of the presence or absence of the *lac* genes, suggesting multiple horizontal transfer events occurred to give rise to the Lac + isolates. Observed STs among the *S. enterica* subsp. *enterica* isolates include ST14 (Senftenberg), ST17 (Indiana), ST316 (Montevideo), and ST319 (Tennessee). The Lac- closed genome strains Enteritidis P125109 (ST11), Typhimurium LT2 (ST19), and Typhi CT18 (ST2) were included in the analysis for reference. Among the *S. enterica* subsp. *diarizonae* strains, isolate 50267 was found to possess ST1261, while isolate 50268 has a variant of ST432 due to a single T to C synonymous transition in *sucA*. The *S. enterica* subsp. *arizonae* closed genome strain RSK2980 was also included for reference as it rooted the MLST phylogeny. RSK2980 has a novel ST comprised of previously observed alleles. Its allele profile of *aroC*65, *dnaN*25, *hemD*29, *hisD*24, *purE*20, *sucA*50, and *thrA*497 is most similar to ST1430, differing by 7 polymorphic sites (1 in *dnaN*, 5 in *hisD*, and 1 in *thrA*).

## Discussion

Strains of *S. enterica* are commonly regarded as non-lactose fermenting pathogens; however a small percentage of isolates have acquired the *lac* region which can lead to confusion from a diagnostic standpoint. The *lac* operon has been well studied in *E. coli* and is known to be chromosomally located. However it has been noted some time ago that the *lac* operon is carried on a plasmid in some *Enterobacteriaceae* species including *S. enterica* Typhimurium and Oranienburg isolates [12]. Subsequent to that report, it was determined that the *lac* operon was located on plasmids harbored in *S. enterica* Typhi isolated from an individual with typhoid fever [14] and *S. enterica* Newport responsible for an outbreak in a nursing home [5]. In contrast, it was shown that the *lac* operon was not carried on a plasmid in an *S. enterica* Indiana isolate [13]. We found that the *lac* region harbored in the Indiana isolate included in this study is also chromosomally located, as are the *lac* regions carried in the *S. enterica* Tennessee genomes. The remaining *S. enterica* subsp. *enterica* isolates in this study possess the *lac* genes on a plasmid similar to *K. pneumoniae* pK29. Interestingly, despite their chromosomal location, the *lac* genes harbored in the Indiana isolate also display high sequence similarity with the *lac* genes found on pK29. Our results revealed other instances of *lac* genes with high sequence similarity but different genomic location (Table 1, Fig. 1); namely, *Enterobacter cloacae* [GenBank:CP009855] and *Pantoea* sp. [GenBank:CP009866] as well as *Klebsiella oxytoca* [GenBank:CP008843] and *Y. enterocolitica* [GenBank:HF571988]. Clearly, the Lac + *S. enterica* isolates harboring a chromosomal *lac* region are not isolates in which the *lac* operon was maintained in the *S. enterica* genome as is thought to be the case for *E. coli*. The most likely explanation for this observation is that *S. enterica* and *E. coli* diverged from a common ancestor and that the *lac* operon was either deleted from *S. enterica* or gained by *E. coli* as suggested by Riley [19].

In fact, we found that two of the *S. enterica* Tennessee isolates in this study possess two *lac* regions each having different sequence homology, flanking organization, and genomic location, none of which is similar to the *lac* region in *E. coli*. Furthermore, our results are consistent with a *lac* region that is highly mobile among *Enterobacteriaceae* as it is almost always flanked by IS elements and this entire region is often inserted in another mobile element, namely a plasmid or chromosomal island. Also, there is a greater correlation between *lac* gene sequence and flanking organization than with genomic location or bacterial species. The combined analysis of genetic organization, MLST, *lac* gene sequence homology, and genomic location in this study clearly demonstrate that *S. enterica* subsp. *enterica* has acquired the *lac* region multiple times through independent events.

The ability to ferment lactose can offer bacteria a growth advantage in environments where lactose is present such as the intestine. Wilkins and Franzese discovered that in a competition experiment utilizing gnotobiotic mice, a lactose-fermenting *E. coli* strain grew to a level ten times greater than the isogenic lactose negative strain [20]. A screen of 552 *S. enterica* isolates from dried milk products or milk-drying plants revealed 15.6 % were Lac + [10], a higher percent than the 0.8 % Lac + *S. enterica* reported in a Centers for Disease Control and Prevention (CDC) survey of 371 cultures [4]. This suggests the readily available lactose in the environment played a selective role for lactose fermenting ability. Interestingly, *S. enterica* Tennessee strain 50261 was isolated from an environment containing dry milk. The other Tennessee isolates in this work were isolated from peanut butter; however source information is not available for many of the isolates in this study so it is unclear how many others may have been associated with a lactose-rich environment.

While lactose fermentation could provide a fitness advantage to *S. enterica* in the environment outside the vertebrate host and in the host gut, it has been reported that the *lacI* and *lacA* genes may act as antivirulence factors by attenuating proliferation inside macrophages or invasion of epithelial cells, respectively, in the host [21–23]. Eswarappa and colleagues demonstrated that, inside macrophages, expression of *lacI* from a plasmid in an *S. enterica* Typhimurium strain missing the *lac* operon causes down regulation of three genes on the SPI-2 pathogenicity island [21]. However, others found no influence on invasion of epithelial cells by a Typhimurium strain harboring a LacI-expressing plasmid [23]. Rather, they concluded that the *lacA* gene, to the same extent as the entire *lac* operon, significantly inhibited transcription of four flagellar genes, thus decreasing epithelial cell invasion [23]. This is consistent with previous experiments utilizing a Typhimurium strain in which it was determined that flagella are required for full invasive potential in a tissue culture invasion assay as well as for a complete inflammatory response in the calf intestine [24].

Although the acquisition of the *lac* operon by a *Salmonella* isolate can be beneficial in some environments, in others it would be a metabolic burden, thus regulation via the LacI protein would be important. Contrary to the assertion that Lac + *S. enterica* strains lack *lacI* [21], we have found that Lac + *S. enterica* carry an intact *lacI* repressor gene. The presence of an intact *lacI* gene in *S. enterica* has also been reported by others [12, 14]. Instead, our results reveal a truncated *lacA* gene in Lac + *S. enterica*. Certainly, Lac + *S. enterica* with intact *lacI*, but truncated *lacA*, have caused gastroenteritis in humans. The truncated *lacA* we noted carried by all the *S. enterica* subsp. *enterica* isolates in this study resulted in a considerably smaller 56 amino acid protein rather than the full length 220 amino acid *E. coli* LacA, therefore it is highly likely it does not exert the effect of repressing the flagellar genes. Macrophage assays may be a superior model for systemic *S. enterica* infections, while epithelial cell invasion assays have been shown to be better models for enteritis in humans since some virulence determinants required for growth at systemic sites such as SPI-2 are less important during an infection localized in the intestinal epithelia [24, 25]. Our genomic characterization results are consistent with the fact that since almost all *S. enterica* subsp. *enterica* are localized to the intestinal tract in the host, *lacI* may not play a role as an antivirulence factor in these strains, while *lacA* appears to be an important antivirulence factor candidate.

## Conclusions

Altogether, our results suggest that the *lac* region is highly mobile among *Enterobacteriaceae* and the Lac + *S. enterica* subsp. *enterica* serovars have acquired the *lac* region through parallel events. The argument could be made that the loss of the *lac* region was originally driven by the adaptation of *S. enterica* as it evolved to become an invasive pathogen. However, some isolates have now reacquired a version of the *lac* region that possesses a truncated *lacA* gene as this would allow the pathogen to utilize lactose when advantageous but not inhibit invasion.

## Methods

### Bacterial strains, culture conditions, and DNA isolation

The 13 *Salmonella enterica* isolates 50259-50265 and 50267-50272 sequenced in this study were kindly provided by Rebecca Dievart. The sequenced isolates along with all other bacteria that were in included in the genetic analysis performed in this study are listed in Table 1. Overnight cultures of the sequenced isolates were grown in Luria broth at 37 °C. The lactose-fermenting phenotype was determined using cultures grown on MacConkey agar plates incubated at 37 °C for 24 h. Genomic DNA was extracted from overnight cultures using the DNeasy Blood and Tissue Kit (Qiagen, Germantown, MD, USA).

### Whole genome sequencing

Sequencing libraries were prepared from genomic DNA with the TruSeq DNA Sample Prep Kit (Illumina, San Diego, CA, USA) and sequenced on the Illumina MiSeq Platform, generating paired-end 250 bp reads in sufficient quantity to provide between 42X and 303X coverage for each genome. Raw reads were trimmed and draft genome sequences were assembled *de novo* with CLC Genomics Workbench v6.5.1 or v7.0.3 (CLC bio, Boston, MA, USA).

### Molecular serotyping

The molecular serotypes of the *S. enterica* isolates were determined from the draft genomes by BLAST analysis using the *wzx*, *wzy*, *wzm*, *wzt*, *wbbE*, *wbbF*, *wbaV*, *weiD*, *oac*, *fliC*, *fljB*, and *flpA* loci.

### Characterization of the lac region and location in genome

The *lac* genes were found in the draft genomes by BLAST using the sequence of the *E. coli* strain K-12 MG1655 operon [GenBank:U00096, locus tags b0342-b0345] as a query. In most cases the entire *lac* operon was contained on one contig; otherwise two contigs were bioinformatically joined to obtain the entire *lac* operon sequence. The joined sequence was then verified by mapping the reads onto the joined sequence using CLC Genomics Workbench (CLC bio). The sequence regions flanking the operons were characterized by BLAST analysis. To determine the location of the *lac* operons within the genome, the *lac* region was used as a BLAST query, followed by identification of the flanking genes by BLAST. The locations of *lac* operon regions in bacterial species with the highest sequence identity were examined for similarity with the *S. enterica* genomes in this study.

### Phylogenetic analyses

DNA sequences of the *lacIZY* region (~5.7 kb) of the operons were combined with those of selected *lac* + bacterial species from GenBank and aligned with the ClustalW algorithm using the MegAlign module of the Lasergene software package (DNAStar Inc., Madison, WI). A pairwise similarity matrix of the *lacIZY* sequences analyzed is provided as supplemental material. *In silico* multilocus sequence typing (MLST) was performed on seven conserved housekeeping genes (*aroC*, *dnaN*, *hemD*, *hisD*, *purE*, *sucA*, and *thrA*) [26] through extraction of the relevant sequences from the draft genome assemblies of the strains investigated in this study. These sequences, along with those of selected *Salmonella* isolates from GenBank (AE006468, AL513382, AM933172, CP000880, CP007505, CP007530, AHUY01000000, AOZC01000000, and AYDO01000000) were concatenated for phylogenetic analysis. Allele and sequence type designations were determined via the *Salmonella* MLST database website (http://mlst.warwick.ac.uk/mlst/dbs/Senterica). Neighbor-joining trees were constructed using the Kimura two-parameter model of nucleotide substitution with the MEGA3 software [27], and the inferred phylogenies were tested with 500 bootstrap replications for both the *lacIZY* and MLST sequences.

### Nucleotide sequence accession numbers

The draft genome sequences of the *S. enterica* strains 50259, 50260, 50261, 50262, 50263, 50264, 50265, 50267, 50268, 50269, 50270, 50271, and 50272 were deposited at DDBJ/EMBL/GenBank under accession nos. JZTJ00000000, JZTK00000000, JZTL00000000, JZTM00000000, JZTN00000000, JZTO00000000, JZTP00000000, JZTQ00000000, JZTR00000000, JZTS00000000, JZTT00000000, JZTU00000000 and JZTV00000000, respectively.

## Additional file

Additional file 1: Figure S1. Pairwise similarity matrix of the lacIZY sequences analyzed in this study. This matrix was generated by calculating the pairwise proportion distances in MEGA3 and then converting the resulting differences (p) to similarities (1-p). Values are color-coded from high to low on a green to red scale. (PDF 72 kb)
