# Supplementary material for: Acquisition of the lac operon by Salmonella enterica
Source: BMC Microbiol. 2015 Aug 25;15:173. doi: 10.1186/s12866-015-0511-8 (PMC4549013; doi:10.1186/s12866-015-0511-8)
Supplement: Additional file 1: Figure S1. — Pairwise similarity matrix of the lacIZY sequences analyzed in this study. This matrix was generated by calculating the pairwise proportion distances in MEGA3 and then converting the resulting differences (p) to similarities (1-p). Values are color-coded from high to low on a green to red scale. (PDF 72 kb) [file 12866_2015_511_MOESM1_ESM.pdf]

Order	Accession no.	Isolate	[01]	[02]	[03]	[04]	[05]	[06]	[07]	[08]	[09]	[10]	[11]	[12]	[13]	[14]	[15]	[16]	[17]	[18]	[19]	[20]	[21]	[22]	[23]	[24]	[25]	[26]	[27]	[28]	[29]	[30]	[31]	[32]	[33]	[34]	[35]	[36]	[37]	[38]	[39]	[40]
[01]	EF382672	K. pneumoniae pK29		0.9996	0.9998	0.9998	0.9998	0.9998	0.9998	0.9998	0.9996	0.9996	0.9996	0.9989	0.9989	0.9945	0.9943	0.9943	0.9943	0.9943	0.9943	0.9935	0.9927	0.9931	0.9941	0.9941	0.9941	0.9941	0.9814	0.9788	0.9827	0.9833	0.9892	0.9894	0.9726	0.9685	0.9703	0.9727	0.9735	0.7315	0.7322	0.7318
[02]	JZTO00000000	S. enterica Senftenberg 50264	0.9996		0.9998	0.9998	0.9998	0.9998	0.9998	0.9998	0.9996	0.9996	0.9996	0.9989	0.9989	0.9945	0.9943	0.9943	0.9943	0.9943	0.9943	0.9935	0.9927	0.9931	0.9941	0.9941	0.9941	0.9941	0.9814	0.9788	0.9827	0.9833	0.9892	0.9894	0.9726	0.9685	0.9703	0.9727	0.9735	0.7315	0.7322	0.7318
[03]	JZTM00000000	S. enterica Montevideo 50262	0.9998	0.9998		1	1	1	1	1	0.9998	0.9998	0.9998	0.9991	0.9991	0.9947	0.9945	0.9945	0.9945	0.9945	0.9945	0.9936	0.9929	0.9933	0.9943	0.9943	0.9943	0.9943	0.9816	0.9789	0.9829	0.9835	0.9894	0.9896	0.9727	0.9687	0.9704	0.9729	0.9736	0.7315	0.7322	0.7318
[04]	JZTN00000000	S. enterica Senftenberg 50263	0.9998	0.9998	1		1	1	1	1	0.9998	0.9998	0.9998	0.9991	0.9991	0.9947	0.9945	0.9945	0.9945	0.9945	0.9945	0.9936	0.9929	0.9933	0.9943	0.9943	0.9943	0.9943	0.9816	0.9789	0.9829	0.9835	0.9894	0.9896	0.9727	0.9687	0.9704	0.9729	0.9736	0.7315	0.7322	0.7318
[05]	JZTP00000000	S. enterica Senftenberg 50265	0.9998	0.9998	1	1		1	1	1	0.9998	0.9998	0.9998	0.9991	0.9991	0.9947	0.9945	0.9945	0.9945	0.9945	0.9945	0.9936	0.9929	0.9933	0.9943	0.9943	0.9943	0.9943	0.9816	0.9789	0.9829	0.9835	0.9894	0.9896	0.9727	0.9687	0.9704	0.9729	0.9736	0.7315	0.7322	0.7318
[06]	JZTT00000000	S. enterica Montevideo 50270	0.9998	0.9998	1	1	1		1	1	0.9998	0.9998	0.9998	0.9991	0.9991	0.9947	0.9945	0.9945	0.9945	0.9945	0.9945	0.9936	0.9929	0.9933	0.9943	0.9943	0.9943	0.9943	0.9816	0.9789	0.9829	0.9835	0.9894	0.9896	0.9727	0.9687	0.9704	0.9729	0.9736	0.7315	0.7322	0.7318
[07]	JZTU00000000	S. enterica Senftenberg 50271	0.9998	0.9998	1	1	1	1		1	0.9998	0.9998	0.9998	0.9991	0.9991	0.9947	0.9945	0.9945	0.9945	0.9945	0.9945	0.9936	0.9929	0.9933	0.9943	0.9943	0.9943	0.9943	0.9816	0.9789	0.9829	0.9835	0.9894	0.9896	0.9727	0.9687	0.9704	0.9729	0.9736	0.7315	0.7322	0.7318
[08]	JZTV00000000	S. enterica Senftenberg 50272	0.9998	0.9998	1	1	1	1	1		0.9998	0.9998	0.9998	0.9991	0.9991	0.9947	0.9945	0.9945	0.9945	0.9945	0.9945	0.9936	0.9929	0.9933	0.9943	0.9943	0.9943	0.9943	0.9816	0.9789	0.9829	0.9835	0.9894	0.9896	0.9727	0.9687	0.9704	0.9729	0.9736	0.7315	0.7322	0.7318
[09]	JZTS00000000	S. enterica Indiana 50269	0.9996	0.9996	0.9998	0.9998	0.9998	0.9998	0.9998	0.9998		1	1	0.9993	0.9993	0.9949	0.9947	0.9947	0.9947	0.9947	0.9947	0.9938	0.9931	0.9935	0.9945	0.9945	0.9945	0.9945	0.9818	0.9791	0.9831	0.9836	0.9896	0.9897	0.9729	0.9688	0.9706	0.9731	0.9738	0.7317	0.7324	0.732
[10]	CP006731	C. sakazakii CMCC 45402	0.9996	0.9996	0.9998	0.9998	0.9998	0.9998	0.9998	0.9998	1		1	0.9993	0.9993	0.9949	0.9947	0.9947	0.9947	0.9947	0.9947	0.9938	0.9931	0.9935	0.9945	0.9945	0.9945	0.9945	0.9818	0.9791	0.9831	0.9836	0.9896	0.9897	0.9729	0.9688	0.9706	0.9731	0.9738	0.7317	0.7324	0.732
[11]	CP010377	E. cloacae 34983	0.9996	0.9996	0.9998	0.9998	0.9998	0.9998	0.9998	0.9998	1	1		0.9993	0.9993	0.9949	0.9947	0.9947	0.9947	0.9947	0.9947	0.9938	0.9931	0.9935	0.9945	0.9945	0.9945	0.9945	0.9818	0.9791	0.9831	0.9836	0.9896	0.9897	0.9729	0.9688	0.9706	0.9731	0.9738	0.7317	0.7324	0.732
[12]	CP004091	C. sakazakii SP291	0.9989	0.9989	0.9991	0.9991	0.9991	0.9991	0.9991	0.9991	0.9993	0.9993	0.9993		0.9996	0.9949	0.9947	0.9947	0.9947	0.9947	0.9947	0.9938	0.9931	0.9935	0.9945	0.9945	0.9945	0.9945	0.9811	0.9784	0.9827	0.9833	0.9896	0.9897	0.9726	0.9685	0.9703	0.9727	0.9735	0.7317	0.7324	0.732
[13]	CP001918	E. cloacae ATCC 13047	0.9989	0.9989	0.9991	0.9991	0.9991	0.9991	0.9991	0.9991	0.9993	0.9993	0.9993	0.9996		0.9949	0.9947	0.9947	0.9947	0.9947	0.9947	0.9938	0.9931	0.9935	0.9945	0.9945	0.9945	0.9945	0.9811	0.9784	0.9827	0.9833	0.9896	0.9897	0.9726	0.9685	0.9703	0.9727	0.9735	0.7313	0.732	0.7317
[14]	CP008842	K. oxytoca pKOXM1A	0.9945	0.9945	0.9947	0.9947	0.9947	0.9947	0.9947	0.9947	0.9949	0.9949	0.9949	0.9949	0.9949		0.9998	0.9998	0.9998	0.9998	0.9998	0.9988	0.9979	0.9982	0.9918	0.9918	0.9918	0.9918	0.9858	0.9832	0.9797	0.9803	0.9858	0.986	0.9685	0.9667	0.9683	0.9697	0.9704	0.7318	0.7324	0.732
[15]	CP000648	K. pneumoniae pKPN3	0.9943	0.9943	0.9945	0.9945	0.9945	0.9945	0.9945	0.9945	0.9947	0.9947	0.9947	0.9947	0.9947	0.9998		1	1	1	1	0.9989	0.9981	0.9984	0.9917	0.9917	0.9917	0.9917	0.986	0.9834	0.9799	0.9804	0.986	0.9862	0.9687	0.9669	0.9685	0.9699	0.9706	0.7318	0.7324	0.732
[16]	CP006657	K. pneumoniae p1	0.9943	0.9943	0.9945	0.9945	0.9945	0.9945	0.9945	0.9945	0.9947	0.9947	0.9947	0.9947	0.9947	0.9998	1		1	1	1	0.9989	0.9981	0.9984	0.9917	0.9917	0.9917	0.9917	0.986	0.9834	0.9799	0.9804	0.986	0.9862	0.9687	0.9669	0.9685	0.9699	0.9706	0.7318	0.7324	0.732
[17]	CP006927	K. pneumoniae p30660_1	0.9943	0.9943	0.9945	0.9945	0.9945	0.9945	0.9945	0.9945	0.9947	0.9947	0.9947	0.9947	0.9947	0.9998	1	1		1	1	0.9989	0.9981	0.9984	0.9917	0.9917	0.9917	0.9917	0.986	0.9834	0.9799	0.9804	0.986	0.9862	0.9687	0.9669	0.9685	0.9699	0.9706	0.7318	0.7324	0.732
[18]	JN233704	K. pneumoniae pKPN-IT	0.9943	0.9943	0.9945	0.9945	0.9945	0.9945	0.9945	0.9945	0.9947	0.9947	0.9947	0.9947	0.9947	0.9998	1	1	1		1	0.9989	0.9981	0.9984	0.9917	0.9917	0.9917	0.9917	0.986	0.9834	0.9799	0.9804	0.986	0.9862	0.9687	0.9669	0.9685	0.9699	0.9706	0.7318	0.7324	0.732
[19]	JX442974	K. pneumoniae pKN-LS6	0.9943	0.9943	0.9945	0.9945	0.9945	0.9945	0.9945	0.9945	0.9947	0.9947	0.9947	0.9947	0.9947	0.9998	1	1	1	1		0.9989	0.9981	0.9984	0.9917	0.9917	0.9917	0.9917	0.986	0.9834	0.9799	0.9804	0.986	0.9862	0.9687	0.9669	0.9685	0.9699	0.9706	0.7318	0.7324	0.732
[20]	FO834905	K. pneumoniae pKP52.145_II	0.9935	0.9935	0.9936	0.9936	0.9936	0.9936	0.9936	0.9936	0.9938	0.9938	0.9938	0.9938	0.9938	0.9988	0.9989	0.9989	0.9989	0.9989	0.9989		0.9977	0.9981	0.9913	0.9913	0.9913	0.9913	0.9857	0.983	0.9799	0.9804	0.9858	0.9862	0.9681	0.9667	0.9681	0.9701	0.9703	0.7318	0.7324	0.732
[21]	JX424424	K. pneumoniae pKPN_CZ	0.9927	0.9927	0.9929	0.9929	0.9929	0.9929	0.9929	0.9929	0.9931	0.9931	0.9931	0.9931	0.9931	0.9979	0.9981	0.9981	0.9981	0.9981	0.9981	0.9977		0.9986	0.9901	0.9901	0.9901	0.9901	0.9848	0.9821	0.979	0.9795	0.9851	0.985	0.9674	0.9658	0.9678	0.9694	0.9699	0.7318	0.732	0.7317
[22]	KF719971	K. pneumoniae pKP007	0.9931	0.9931	0.9933	0.9933	0.9933	0.9933	0.9933	0.9933	0.9935	0.9935	0.9935	0.9935	0.9935	0.9982	0.9984	0.9984	0.9984	0.9984	0.9984	0.9981	0.9986		0.9904	0.9904	0.9904	0.9904	0.9851	0.9825	0.979	0.9795	0.9851	0.9853	0.9674	0.9658	0.9674	0.969	0.9699	0.7322	0.7327	0.7324
[23]	JZTJ00000000	S. enterica Tennessee 50259	0.9941	0.9941	0.9943	0.9943	0.9943	0.9943	0.9943	0.9943	0.9945	0.9945	0.9945	0.9945	0.9945	0.9918	0.9917	0.9917	0.9917	0.9917	0.9917	0.9913	0.9901	0.9904		1	1	1	0.9815	0.9789	0.9868	0.9874	0.9874	0.9878	0.9704	0.9673	0.9693	0.9716	0.9725	0.7317	0.7328	0.7322
[24]	JZTK00000000	S. enterica Tennessee 50260 lac1	0.9941	0.9941	0.9943	0.9943	0.9943	0.9943	0.9943	0.9943	0.9945	0.9945	0.9945	0.9945	0.9945	0.9918	0.9917	0.9917	0.9917	0.9917	0.9917	0.9913	0.9901	0.9904	1		1	1	0.9815	0.9789	0.9868	0.9874	0.9874	0.9878	0.9704	0.9673	0.9693	0.9716	0.9725	0.7317	0.7328	0.7322
[25]	JZTL00000000	S. enterica Tennessee 50261 lac1	0.9941	0.9941	0.9943	0.9943	0.9943	0.9943	0.9943	0.9943	0.9945	0.9945	0.9945	0.9945	0.9945	0.9918	0.9917	0.9917	0.9917	0.9917	0.9917	0.9913	0.9901	0.9904	1	1		1	0.9815	0.9789	0.9868	0.9874	0.9874	0.9878	0.9704	0.9673	0.9693	0.9716	0.9725	0.7317	0.7328	0.7322
[26]	AHUY01000000	S. enterica Tennessee 4535	0.9941	0.9941	0.9943	0.9943	0.9943	0.9943	0.9943	0.9943	0.9945	0.9945	0.9945	0.9945	0.9945	0.9918	0.9917	0.9917	0.9917	0.9917	0.9917	0.9913	0.9901	0.9904	1	1	1		0.9815	0.9789	0.9868	0.9874	0.9874	0.9878	0.9704	0.9673	0.9693	0.9716	0.9725	0.7317	0.7328	0.7322
[27]	CP008843	K. oxytoca pKOXM1B	0.9814	0.9814	0.9816	0.9816	0.9816	0.9816	0.9816	0.9816	0.9818	0.9818	0.9818	0.9811	0.9811	0.9858	0.986	0.986	0.986	0.986	0.986	0.9857	0.9848	0.9851	0.9815	0.9815	0.9815	0.9815		0.9963	0.9838	0.9843	0.9727	0.9733	0.9605	0.9612	0.965	0.9635	0.9642	0.7318	0.7322	0.7318
[28]	HF571988	Y. enterocolitica YE53/03	0.9788	0.9788	0.9789	0.9789	0.9789	0.9789	0.9789	0.9789	0.9791	0.9791	0.9791	0.9784	0.9784	0.9832	0.9834	0.9834	0.9834	0.9834	0.9834	0.983	0.9821	0.9825	0.9789	0.9789	0.9789	0.9789	0.9963		0.9815	0.982	0.9704	0.971	0.96	0.96	0.9644	0.9627	0.9634	0.7311	0.7317	0.7315
[29]	JZTK00000000	S. enterica Tennessee 50260 lac2	0.9827	0.9827	0.9829	0.9829	0.9829	0.9829	0.9829	0.9829	0.9831	0.9831	0.9831	0.9827	0.9827	0.9797	0.9799	0.9799	0.9799	0.9799	0.9799	0.9799	0.979	0.979	0.9868	0.9868	0.9868	0.9868	0.9838	0.9815		0.9995	0.9763	0.9767	0.9646	0.9633	0.9662	0.9673	0.9671	0.7305	0.7319	0.7315
[30]	JZTL00000000	S. enterica Tennessee 50261 lac2	0.9833	0.9833	0.9835	0.9835	0.9835	0.9835	0.9835	0.9835	0.9836	0.9836	0.9836	0.9833	0.9833	0.9803	0.9804	0.9804	0.9804	0.9804	0.9804	0.9804	0.9795	0.9795	0.9874	0.9874	0.9874	0.9874	0.9843	0.982	0.9995		0.9769	0.9772	0.9651	0.9639	0.9667	0.9678	0.9676	0.731	0.7324	0.7321
[31]	CP009855	E. cloacae pENT-22e	0.9892	0.9892	0.9894	0.9894	0.9894	0.9894	0.9894	0.9894	0.9896	0.9896	0.9896	0.9896	0.9896	0.9858	0.986	0.986	0.986	0.986	0.986	0.9858	0.9851	0.9851	0.9874	0.9874	0.9874	0.9874	0.9727	0.9704	0.9763	0.9769		0.9984	0.9777	0.9699	0.9724	0.9752	0.9761	0.7321	0.7328	0.7324
[32]	CP009866	Pantoea sp. PSNIH2	0.9894	0.9894	0.9896	0.9896	0.9896	0.9896	0.9896	0.9896	0.9897	0.9897	0.9897	0.9897	0.9897	0.986	0.9862	0.9862	0.9862	0.9862	0.9862	0.9862	0.985	0.9853	0.9878	0.9878	0.9878	0.9878	0.9733	0.971	0.9767	0.9772	0.9984		0.9775	0.9699	0.9724	0.9752	0.9759	0.7326	0.7333	0.733
[33]	BA000007	E. coli Sakai	0.9726	0.9726	0.9727	0.9727	0.9727	0.9727	0.9727	0.9727	0.9729	0.9729	0.9729	0.9726	0.9726	0.9685	0.9687	0.9687	0.9687	0.9687	0.9687	0.9681	0.9674	0.9674	0.9704	0.9704	0.9704	0.9704	0.9605	0.96	0.9646	0.9651	0.9777	0.9775		0.9713	0.9754	0.974	0.9751	0.7326	0.7335	0.7334
[34]	AE014075	E. coli CFT073	0.9685	0.9685	0.9687	0.9687	0.9687	0.9687	0.9687	0.9687	0.9688	0.9688	0.9688	0.9685	0.9685	0.9667	0.9669	0.9669	0.9669	0.9669	0.9669	0.9667	0.9658	0.9658	0.9673	0.9673	0.9673	0.9673	0.9612	0.96	0.9633	0.9639	0.9699	0.9699	0.9713		0.9752	0.9722	0.9745	0.7372	0.7377	0.7376
[35]	FN554766	E. coli 042	0.9703	0.9703	0.9704	0.9704	0.9704	0.9704	0.9704	0.9704	0.9706	0.9706	0.9706	0.9703	0.9703	0.9683	0.9685	0.9685	0.9685	0.9685	0.9685	0.9681	0.9678	0.9674	0.9693	0.9693	0.9693	0.9693	0.965	0.9644	0.9662	0.9667	0.9724	0.9724	0.9754	0.9752		0.9768	0.9786	0.7369	0.738	0.7378
[36]	CP003289	E. coli 2011C-3493	0.9727	0.9727	0.9729	0.9729	0.9729	0.9729	0.9729	0.9729	0.9731	0.9731	0.9731	0.9727	0.9727	0.9697	0.9699	0.9699	0.9699	0.9699	0.9699	0.9701	0.9694	0.969	0.9716	0.9716	0.9716	0.9716	0.9635	0.9627	0.9673	0.9678	0.9752	0.9752	0.974	0.9722	0.9768		0.9873	0.7341	0.735	0.7348
[37]	U00096	E. coli K-12 MG1655	0.9735	0.9735	0.9736	0.9736	0.9736	0.9736	0.9736	0.9736	0.9738	0.9738	0.9738	0.9735	0.9735	0.9704	0.9706	0.9706	0.9706	0.9706	0.9706	0.9703	0.9699	0.9699	0.9725	0.9725	0.9725	0.9725	0.9642	0.9634	0.9671	0.9676	0.9761	0.9759	0.9751	0.9745	0.9786	0.9873		0.7357	0.7371	0.7369
[38]	CP000880	S. enterica arizonae RSK2980	0.7315	0.7315	0.7315	0.7315	0.7315	0.7315	0.7315	0.7315	0.7317	0.7317	0.7317	0.7317	0.7313	0.7318	0.7318	0.7318	0.7318	0.7318	0.7318	0.7318	0.7318	0.7322	0.7317	0.7317	0.7317	0.7317	0.7318	0.7311	0.7305	0.731	0.7321	0.7326	0.7326	0.7372	0.7369	0.7341	0.7357		0.9799	0.9798
[39]	JZTQ00000000	S. enterica diarizonae 50267	0.7322	0.7322	0.7322	0.7322	0.7322	0.7322	0.7322	0.7322	0.7324	0.7324	0.7324	0.7324	0.732	0.7324	0.7324	0.7324	0.7324	0.7324	0.7324	0.7324	0.732	0.7327	0.7328	0.7328	0.7328	0.7328	0.7322	0.7317	0.7319	0.7324	0.7328	0.7333	0.7335	0.7377	0.738	0.735	0.7371	0.9799		0.9984
[40]	JZTR00000000	S. enterica diarizonae 50268	0.7318	0.7318	0.7318	0.7318	0.7318	0.7318	0.7318	0.7318	0.732	0.732	0.732	0.732	0.7317	0.732	0.732	0.732	0.732	0.732	0.732	0.732	0.7317	0.7324	0.7322	0.7322	0.7322	0.7322	0.7318	0.7315	0.7315	0.7321	0.7324	0.733	0.7334	0.7376	0.7378	0.7348	0.7369	0.9798	0.9984	

Figure S1. Pairwise similarity matrix of the *lacIZY* sequences analyzed in this study. This matrix was generated by calculating the pairwise proportion distances in MEGA3 and then converting the resulting differences (p) to similarities (1-p). Values are color-coded from high to low on a green to red scale.
